# Association between *HSPA8* Gene Variants and Ischemic Stroke: A Pilot Study Providing Additional Evidence for the Role of Heat Shock Proteins in Disease Pathogenesis

**DOI:** 10.3390/genes14061171

**Published:** 2023-05-27

**Authors:** Ksenia A. Kobzeva, Maria O. Soldatova, Tatiana A. Stetskaya, Vladislav O. Soldatov, Alexey V. Deykin, Maxim B. Freidin, Marina A. Bykanova, Mikhail I. Churnosov, Alexey V. Polonikov, Olga Y. Bushueva

**Affiliations:** 1 Laboratory of Genomic Research, Research Institute for Genetic and Molecular Epidemiology, Kursk State Medical University, 305041 Kursk, Russia; 2 Laboratory of Statistical Genetics and Bioinformatics, Research Institute for Genetic and Molecular Epidemiology, Kursk State Medical University, 305041 Kursk, Russia; 3 Laboratory of Genome Editing for Biomedicine and Animal Health, Belgorod State National Research University, 308015 Belgorod, Russia; 4 Department of Pharmacology and Clinical Pharmacology, Belgorod State National Research University, 308015 Belgorod, Russia; 5 Department of Biology, School of Biological and Behavioural Sciences, Queen Mary University of London, London E1 4NS, UK; 6 Laboratory of Population Genetics, Research Institute of Medical Genetics, Tomsk National Research Medical Center, Russian Academy of Science, 634050 Tomsk, Russia; 7 Department of Medical Biological Disciplines, Belgorod State University, 308015 Belgorod, Russia; 8 Department of Biology, Medical Genetics and Ecology, Kursk State Medical University, 305041 Kursk, Russia

**Keywords:** ischemic stroke, chaperones, heat shock proteins, *HSPA8*, *HSC70*, rs10892958, rs1136141, gene-environmental interaction, smoking, fruit and vegetable intake

## Abstract

HSPA8 is involved in many stroke-associated cellular processes, playing a pivotal role in the protein quality control system. Here we report the results of the pilot study aimed at determining whether *HSPA8* SNPs are linked to the risk of ischemic stroke (IS). DNA samples from 2139 Russians (888 IS patients and 1251 healthy controls) were genotyped for tagSNPs (rs1461496, rs10892958, and rs1136141) in the *HSPA8* gene using probe-based PCR. SNP rs10892958 of *HSPA8* was associated with an increased risk (risk allele G) of IS in smokers (OR = 1.37; 95% CI = 1.07–1.77; *p* = 0.01) and patients with low fruit and vegetable consumption (OR = 1.36; 95% CI = 1.14–1.63; *p* = 0.002). SNP rs1136141 of *HSPA8* was also associated with an increased risk of IS (risk allele A) exclusively in smokers (OR = 1.68; 95% CI = 1.23–2.28; *p* = 0.0007) and in patients with a low fruit and vegetable intake (OR = 1.29; 95% CI = 1.05–1.60; *p* = 0.04). Sex-stratified analysis revealed an association of rs10892958 *HSPA8* with an increased risk of IS in males (risk allele G; OR = 1.30; 95% CI = 1.05–1.61; *p* = 0.01). Thus, SNPs rs10892958 and rs1136141 in the *HSPA8* gene represent novel genetic markers of IS.

## 1. Introduction

Ischemic stroke is the most frequent type of brain attack, the leading cause of death worldwide [1]. IS is considered a multifactorial disease due to the involvement of genetic and environmental factors [2]. A lot of studies have been done to evaluate the genetic nature of IS [3,4,5,6,7] and IS-related modifiable risk factors, like hypertension [8,9], atherosclerosis [10,11], thrombosis [12]. However, disclosure of genetic correlates of IS remains an important task on the way to predicting and fighting the disease.

In ischemic conditions, a cell relies on multiple interplaying ensembles preserving macromolecules from degradation caused by oxygen deprivation, acidosis and oxidation. Heat-shock proteins (HSPs) are a type of chaperones that are considered to be the most conservative molecular machines providing homeostasis in ischemic conditions. HSPs encourage the refolding of misfolded or immature proteins and prevent aggregation of misfolded proteins, which are involved in apoptosis, necrosis, and inflammation, which determines their significant role under pathological conditions, in particular ischemia-reperfusion [13]. Accordingly, a large body of evidence has shown HSPs to be among the most impactful players in response to ischemia as well as in recovering after ischemia-reperfusion injury [14].

The *HSPA8* gene encodes a member of the heat shock protein 70 (HSP70) family known as the heat shock cognate 71 kDa protein (HSC70). HSP70 is known for its neuroprotective functions [15,16,17,18], and as well as other members, HSP70 maintains cellular homeostasis [19]. Precise roles of HSC70 include folding and transport of newly synthesised polypeptides and assembly of protein complexes [20,21,22], regulation of mitochondrial import [23], and the ER-associated degradation quality control system [24]. According to our knowledge, no research has looked into the relationship between *HSPA8* genetic variants and IS. Therefore, the objective of our study was to challenge whether polymorphisms of *HSPA8* gene are related to IS risk.

## 2. Materials and Methods

**Figure 1:**
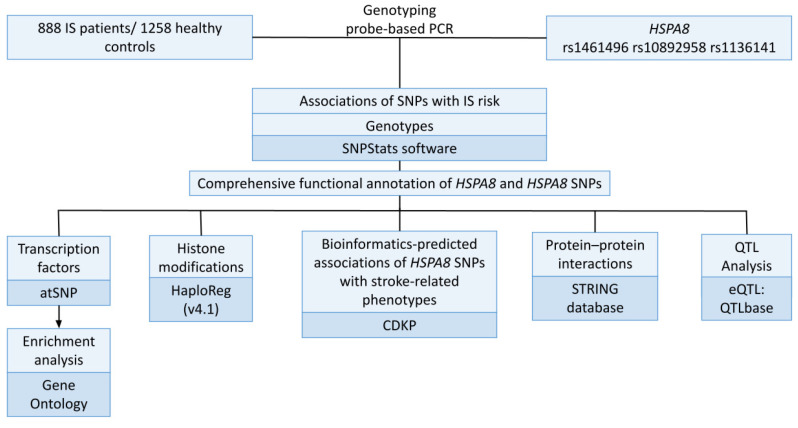
Study design.

Figure 1 depicts the research design’s general layout.

For the study, a total of 2139 unrelated Russians (888 IS patients and 1251 healthy individuals) from Central Russia were enrolled. The study protocol was approved by the Kursk State Medical University’s Ethical Review Committee. All participants provided written informed consent prior to being accepted into the study, with the following inclusion requirements: self-declared Russian ancestry and a birthplace in Central Russia. Table 1 provides the baseline and clinical characteristics of the study cohort.

The participants were enrolled in the study in two time periods: between 2010 and 2012 at the Kursk Emergency Medicine Hospital’s neurology clinics [25] and between 2015 and 2017 at the Regional Vascular Centre of Kursk Regional Clinical Hospital [26]. A team of qualificated neurologists assessed each case. The diagnosis of IS during the acute phase of stroke was confirmed using the findings of brain computed tomography and/or magnetic resonance imaging. The patients were recruited consecutively. Intracerebral hemorrhage, hemodynamic or dissection-related stroke, traumatic brain injury, hepatic or renal failure, autoimmune, oncological, or other disorders that can produce an acute cerebrovascular event were considered exclusion criteria. All of the IS patients had a history of hypertension and were receiving antihypertensive medications.

According to the WHO recommendations, low fruit and vegetable consumption was defined as an intake of less than 400 g per day. Normal consumption of fresh vegetables and fruits was defined as an intake of 400 g or more in 3–4 servings per day (excluding potatoes and other starchy tubers). [27]. The control group was made up of healthy volunteers with normal blood pressure who did not take antihypertensive therapy and did not exhibit any clinical symptoms of cardiovascular, cerebrovascular, or other major diseases. Healthy persons were included in the control group if their systolic blood pressure was less than 130 mm Hg and their diastolic blood pressure was less than 85 mm Hg on at least three different measurements. In the Kursk region, controls were chosen from the hospitals in Kursk during routine medical exams at public institutions and industrial businesses [28,29]. This group was recruited from the same population and during the same period.

The following criteria were used to choose the SNPs: they had to be tagging, have a minor allele frequency of at least 0.05 in the European population, and be distinguished by a high regulatory potential. The bioinformatic tools LD TAG SNP Selection (TagSNP) and SNPinfo Web Server (https://snpinfo.niehs.nih.gov/(accessed on 15 March 2022)), which were utilised to choose SNPs based on the reference haplotypic structure of the Caucasian population (CEU) of the project HapMap, showed that the *HSPA8* (heat shock protein family A (Hsp70) member 8, ID: 3312) gene contains three tag SNPs (rs1461496, rs10892958, and rs1136141). SNPs rs1461496 and rs1136141 are located in the 3 prime UTRs; rs10892958 is located in the intron.

Several bioinformatic approaches were used to examine the *HSPA8* tag SNPs’ regulatory potential. SNP FuncPred reports that the rs10892958 has a Regulatory Potential Score of 0.154 and the rs1136141 has a Regulatory Potential Score of 0.132 (https://snpinfo.niehs.nih.gov/snpinfo/snpfunc.html (accessed on 15 February 2023)) [30].

The RegulomeDB instrument revealed that rs1461496 is characterized by a regulatory coefficient of 3a (TF binding + any motif + DNase peak); rs10892958 and rs1136141 are characterized by a regulatory coefficient of 4 (TF binding + DNase peak) (https://regulomedb.org/regulome-search/ (accessed on 15 February 2023)) [31]. 

According to the HaploReg (v4.1) database, these SNPs have properties such as enhancer histone tags in various tissues (rs1461496), regions of hypersensitivity for DNAse 1 (rs1461496, rs10892958, rs1136141), binding sites for regulatory proteins (rs10892958, rs1136141), and DNA regulatory motifs (rs10892958, rs1136141) (http://archive.broadinstitute.org/mammals/haploreg/haploreg.php (accessed on 15 February 2023)) [32].

According to information from the NCBI source (https://www.ncbi.nlm.nih.gov/snp/ (accessed on 15 February 2023)), these genetic variants are identified by an average frequency of the minor alleles in European populations of >0.05. As a result, all three SNPs were chosen for our investigation, which satisfied the requirements for study inclusion.

### 2.1. Genetic Analysis

The Laboratory of Genomic Research at the Research Institute for Genetic and Molecular Epidemiology of Kursk State Medical University (Kursk, Russia) performed genotyping. Up to 5 mL of venous blood from each participant was collected from a cubital vein, put into EDTA-coated tubes, and kept at −20 °C until it was processed. Defrosted blood samples were used to extract genomic DNA using the standard methods of phenol/chloroform extraction and ethanol precipitation. The purity, quality, and concentration of the extracted DNA solution were assessed using a NanoDrop spectrophotometer (Thermo Fisher Scientific, Waltham, MA, USA).

Genotyping of the SNPs was done using allele-specific probe-based polymerase chain reaction (PCR) according to the protocols designed in the Laboratory of Genomic Research. The Primer3 software was used for primer design [33]. Table S1 lists the primers and probes used for genotyping. A real-time PCR procedure was performed in a 25 mL reaction solution containing 1.5 units of Hot Start Taq DNA polymerase (Biolabmix, Novosibirsk, Russia), approximately 1 μg of DNA, 0.25 μM each of the primers 0.25 μM each primer, 250 μM dNTPs, 3.0 mM MgCl_2_ (for rs1136141 and rs1461496), 4.5 mM MgCl_2_ (for rs10892958); 1×PCR buffer (67 mM Tris-HCl, pH 8.8, 16.6 mM (NH_4_)_2_SO_4_, 0.01% Tween-20). The PCR procedure comprised an initial denaturation for 10 minutes at 95°C, followed by 39 cycles of 92 °C for 30 s and 65 °C, 61.5 °C, 51 °C for 1 min (for rs10892958, rs1136141 and rs1461496 respectively). Figures S1–S3 show allelic discrimination plots for *HSPA8* assays designed for this study. 10% of the DNA samples were genotyped twice, blinded to the case-control status, in order to assure quality control. Over 99% of the data were concordant. 

### 2.2. Statistical and Bioinformatic Analysis

The statistical power for the study was determined using the genetic association study power calculator, a tool available online at http://csg.sph.umich.edu/abecasis/gas_power_calculator/ (accessed on 16 February 2023). Given a sample size of 888 cases and 1251 controls, an association study between the *HSPA8* gene polymorphisms and IS risk might identify a genotype relative risk of 1.20–1.32 assuming 0.80 power and a 5% type I error (α = 0.05).

The STATISTICA software (v13.3, Santa Clara, California, USA) was used to conduct all statistical analyses. The Shapiro-Wilk test was used to determine the normality of the distribution of quantitative data. The median (Me) and first and third quartiles [Q1 and Q3] were used to express the biochemical parameters and body mass index because they deviated from the normal distribution. The Pearson’s chi-squared test with Yates’s continuity correction was used to determine the statistical significance of differences for categorical variables. 

Using Fisher’s exact test, the genotype distributions were evaluated for Hardy-Weinberg equilibrium compliance. Using the SNPStats software (https://www.snpstats.net/start.htm (accessed on 18 January 2023)), the genotype frequencies in the study groups and their relationships to disease risk were analyzed [34]. For the analysis of associations of genotypes, additive models were used. The associations of genotypes in the whole group of IS patients and controls were adjusted for age, gender, and smoking status. When the environmental risk factor in the control group was unknown, relationships were examined based on whether the risk factor was present or absent in the IS group relative to the entire control group. The Bonferroni correction was also used in this instance. 

The functional effects of *HSPA8* SNPs were examined using the bioinformatics resources listed below:

- The expression quantitative trait loci (eQTLs) in the brain, whole blood, and blood vessels have been evaluated using the bioinformatic tool QTLbase (http://www.mulinlab.org/qtlbase/index.html (accessed on 21 February 2023)) [35].
- The STRING database’s bioinformatic tools were utilised to analyse the main functional partners of HSPA8 (https://string-db.org/ (accessed on 21 February 2023)) [36]. Additionally, the STRING database was used to assess biological processes and molecular functions data describing interactions between HSPA8 and its functionally significant partner proteins. For the interpretation of interactions only experimentally confirmed data was used.
- The effect of *HSPA8* SNPs on the binding of transcription factors (TFs) to DNA was assessed using the atSNP Function Prediction online tool (http://atsnp.biostat.wisc.edu/search (accessed on 21 February 2023)) [37]. Based on a positional weight matrix-based calculation of the impact of SNPs on how well TFs interact with DNA, certain TFs were added.
- The online Gene Ontology tool was used to conduct the subsequent study of the potential joint involvement of TFs linked with the reference and SNP alleles in overrepresented biological processes that are related to the mechanisms of IS (http://geneontology.org/ (accessed on 21 February 2023)) [38]. As functional groups, we employed biological processes governed by transcription factors connected to *HSPA8* SNPs.
- HaploReg (v4.1), a bioinformatics tool (http://archive.broadinstitute.org/mammals/haploreg/haploreg.php (accessed on 20 February 2023)) was used to evaluate the relationships between *HSPA8* SNPs and the following histone modifications that mark promoters and enhancers: acetylation of the lysine residues at positions 27 and 9 of the histone H3 protein, as well as mono-methylation at position 4 of the histone H3 protein (H3K4me1) and tri-methylation at position 4 of the histone H3 protein (H3K4me3). Additionally, this tool has been employed to examine the localization of SNPs in DNase hypersensitive areas, regulatory motif regions, and locations that bind to regulatory proteins [32].
- The interpretation of environment-associated correlates of *HSPA8* polymorphism was done using the Comparative Toxicogenomics Database (CTD) resource at http://ctdbase.org (accessed on 24 February 2023) [39]. Based on data gathered from internationally published scientific studies, CTD offers the capability to investigate particular interactions between genes and chemicals in vertebrates and invertebrates. Using this method, bidirectional interactions comprising a single chemical and a single gene or protein were examined.
- The Cerebrovascular Disease Knowledge Portal (CDKP) is available at https://cd.hugeamp.org/ (accessed on 24 February 2023) was employed for a bioinformatic investigation of the relationships between *HSPA8* SNPs and stroke-related traits, intermediate phenotypes, and risk factors for IS (such as blood pressure, heart rate, etc.) [40].

## 3. Results

### 3.1. Bioinformatic Analysis of the HSPA8 Gene

**Figure 2:**
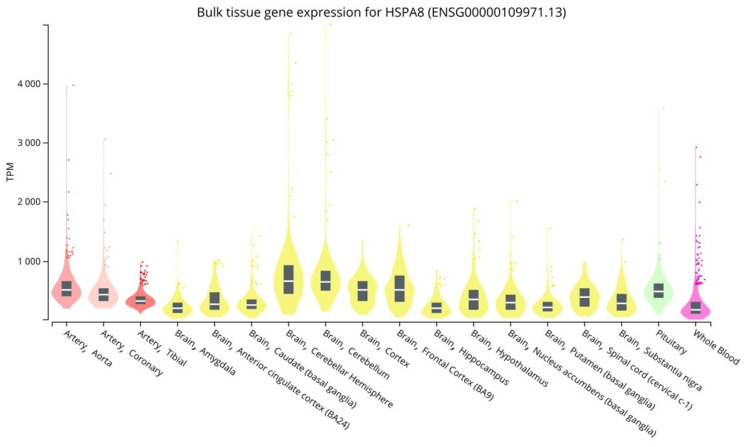
*HSPA8* expression levels in vessels, brain, and peripheral blood (https://gtexportal.org/home/ (accessed on 15 February 2023)).

Brain tissues, blood vessels, and whole blood have high levels of *HSPA8* gene expression. *HSPA8* gene expression levels (MeTPM) range from 197.4 to 662.8 in brain tissues, from 327.8 to 509.0 in blood vessels, and are 177.0 in whole blood (Figure 2).

#### Protein–Protein Interactions

**Figure 3:**
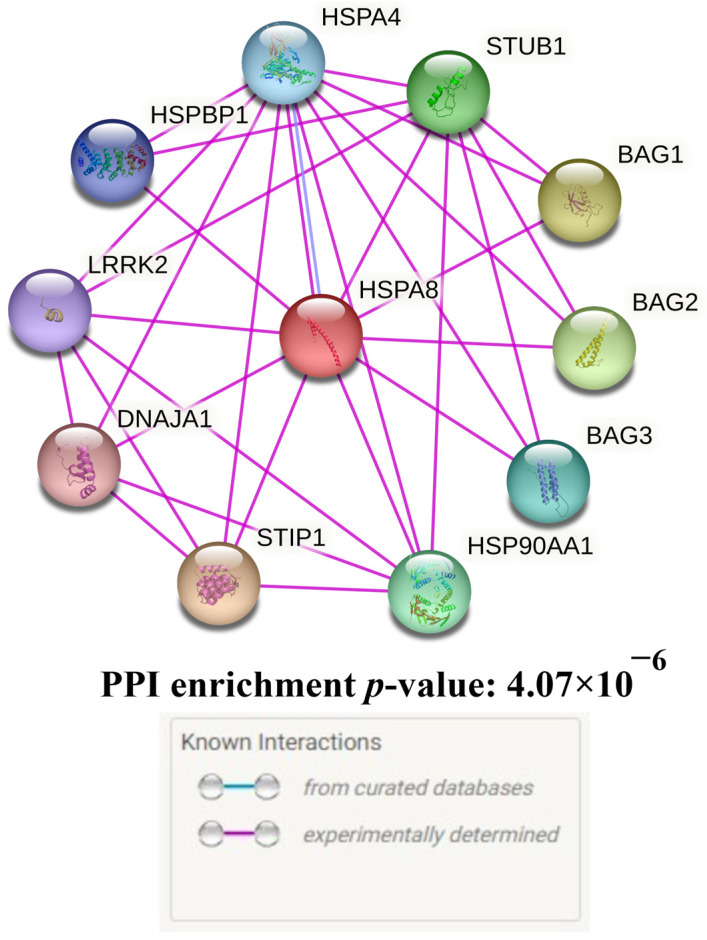
Predicted functional partners of HSPA8.

Ten proteins having the most prominent interactions with HSPA8 were found after searching for the primary functional partners of HSPA8 using the STRING database data: STIP1, BAG1, BAG2, STUB1, HSP90AA1, BAG3, HSPA4, HSPBP1, LRRK2, DNAJA1 (Figure 3, Table S2).

HSPA8 and its key functional partners participate in 40 GO, predominantly reflecting proteostasis (for example, “protein folding” (GO = 0006457, FDR = 8.40 × 10^−8^), “response to unfolded protein” (GO = 0006986, FDR = 8.04 × 10^−7^), “regulation of protein ubiquitination” (GO = 0031396, FDR = 1.68 × 10^−6^), “regulation of protein stability” (GO = 0031647, FDR = 8.40 × 10^−6^), and “regulation of protein ubiquitination” (GO = 0031396, FDR = 0.004)), heat shock (for example, “regulation of cellular response to heat” (GO = 1900034, FDR = 1.50 × 10^−6^) and “response to heat” (GO = 0009408, FDR = 1.70 × 10^−4^)) and autophagy (“chaperone-mediated autophagy” (GO = 0061684, FDR = 1.71 × 10^−7^) and “autophagy” (GO: 0006914, FDR = 1.70 × 10^−4^)) (the full list of biological processes of HSPA8 and its main functional partners is presented in Table S3).

The presented data provide additional confirmation of the significant role of HSPA8 in protein homeostasis, heat shock, and autophagy. Additionally, the data confirm the potentially high pathogenetic significance of this gene in relation to the risk of developing IS.

### 3.2. HSPA8 SNPs and the Ischemic Stroke Risk: An Analysis of Associations 

Table S4 displays the genotype frequencies of the *HSPA8* variants (rs1461496, rs10892958, and rs1136141) in the study groups. In the control group, the distribution of genotype frequencies for each of the investigated SNPs matched the Hardy-Weinberg equilibrium (*p* > 0.05). The observed heterozygosity for rs1136141 (0.2280) in IS patients, nonetheless, was lower than expected (0.2495); *p* < 0.05 (Table S4). 

There were no links between IS risk and HSPA8 SNPs in the analysis of the entire cohort (Table 2). Following sub-group analysis, it was revealed that rs10892958 was linked to a higher risk of IS only in males (risk allele G; OR = 1.30; 95% CI = 1.05–1.61; *p* = 0.01). The genetic variants rs10892958 (risk allele G; OR = 1.37; 95% CI = 1.07–1.77; *p* = 0.01) and rs1136141 (risk allele A; OR = 1.68; 95% CI = 1.23–2.28; *p* = 7.0 × 10^−4^) were associated with the development of IS only in smokers. It is noteworthy that the associations between rs10892958 (risk allele G; OR = 1.36; 95% CI = 1.14–1.63; *p* = 9.0 × 10^−4^; Pbonf = 0.002) and rs1136141 (risk allele A; OR = 1.29; 95% CI = 1.05–1.60; *p* = 0.02; Pbonf = 0.04) with IS risk were observed only under the condition of low fruit and vegetable intake (Table 2; Table S5). 

### 3.3. Functional Annotation of HSPA8 SNPs

#### 3.3.1. QTL-Effects

Table 3 displays the results of the eQTL analysis for the *HSPA8* SNPs. The QTLbase browser data show that the risk allele G rs10892958 is associated with decreased expression of *HSPA8* in Brain-Hippocampus; risk allele A rs1136141 is related to reduced expression of *HSPA8* and increased expression of *CLMP* in Brain-Hippocampus (Table 3).

#### 3.3.2. Histone Modifications

A further examination revealed the substantial effect of IS-related *HSPA8* SNPs on histone tags. SNPs rs10892958 and rs1136141 are located in the region of DNA binding to H3K4me1 in brain tissues (rs10892958), blood (rs10892958, rs1136141), as well as H3K4me3 in the brain tissues and blood (rs10892958, rs1136141). The effect of these histone tags is increased by the H3K27ac, marking enhancers in blood cells and all brain tissues (rs10892958, rs1136141), as well as the H3K9ac, marking promoters in blood cells and all brain tissues, except the Brain Hippocampus Middle (rs10892958, rs1136141). It is noteworthy that the SNPs rs10892958 and rs1136141 are also localized in DNA regions hypersensitive to DNase-1 in the blood (Table 4).

Moreover, using the resource HaploReg (v4.1), it was found that these SNPs are highly regulated by regulatory proteins: rs1136141 is located in the binding site DNA with 23 regulatory proteins: CMYC, ELF1, ELK4, GTF2B, GTF2F1, HEY1, MXI1, NELFE, NFKB, OCT2, P300, POL2, POL24H8, POL2B, POL2S2, POU2F2, SIN3AK20, TAF1, TAF7, TBP, TCF12, TCF4, YY1. SNP rs10892958 is localized at the DNA binding site with 6 regulatory proteins: CMYC, OCT2, POL2, POL24H8, POU2F2, TAF7.

#### 3.3.3. Analysis of Transcription Factors

The risk allele G rs10892958 of *HSPA8* generates DNA binding sites for 31 TFs that are simultaneously implicated in 6 overrepresented GO regulating oxidative stress and neurogenesis: “regulation of transcription from RNA polymerase II promoter in response to oxidative stress” (GO = 0043619; FDR = 0.02), “glial cell fate commitment” (GO = 0021781; FDR = 0.04), “regulation of oligodendrocyte differentiation” (GO = 0048713; FDR = 0.01), “neuron fate commitment” (GO = 0048663; FDR = 0.0009), “negative regulation of neuron differentiation” (GO = 0045665; FDR = 0.04), and “oligodendrocyte differentiation” (GO = 0048709; FDR = 0.04) (Table S6). Meanwhile, protective allele C rs10892958 *HSPA8* provides DNA binding regions for 53 TFs jointly involved in the regulation of proatherosclerotic mechanisms, inflammation, cell signaling, neurogenesis, and apoptosis: “macrophage derived foam cell differentiation” (GO = 0010742; FDR = 0.0149), “cellular response to transforming growth factor beta stimulus” (GO = 0071560; FDR = 0.02), “cellular response to cytokine stimulus” (GO = 0071345; FDR = 0.008), “negative regulation of interferon-beta production” (GO = 0032688; FDR = 0.035), “glial cell fate commitment” (GO = 0021781; FDR = 0.03), and “regulation of apoptotic process” (GO = 0042981; FDR = 0.01) (Table S6). 

Risk allele A, rs1136141, of *HSPA8* creates DNA binding sites for 43 TFs, which together participate in 5 overrepresented GOs co-controlling “positive regulation of CD8-positive, alpha-beta T cell differentiation” (GO = 0043378; FDR = 0.007), “regulation of blood vessel endothelial cell migration” (GO = 0043535; FDR = 0.04), “positive regulation of angiogenesis” (GO = 0045766; FDR = 0.02), “response to growth factor” (GO = 0070848; FDR = 0.015), and “negative regulation of apoptotic process” (GO = 0043066; FDR = 0.04). Meanwhile, no common GO was defined for 19 TFs, binding with the protective allele G (Table S7).

#### 3.3.4. Bioinformatic Analysis of the Associations of *HSPA8* SNPs with IS-Related Phenotypes

According to the bioinformatic resource CDKP, which combines and analyzes the results of genetic associations from the largest consortiums for the study of cerebrovascular diseases, risk allele A rs1136141 of *HSPA8* is associated with increases in systolic blood pressure, heart rate, peripheral artery disease in ever-smokers and stroke (TOAST, other-determined) (Table 5).

## 4. Discussion

Our study is the first to show that *HSPA8* SNPs are associated with the risk and clinical features of IS, and this relationship is significantly modified by gender and environmental risk factors. SNP rs10892958 (risk allele G) was found to be associated with an increased risk of IS exclusively in males, smokers, and individuals with low fruit and vegetable consumption. SNP rs1136141 was associated with an increased risk of IS only in smokers and on the condition of insufficient fruit and vegetable consumption. 

HSPA8 is a molecular chaperone highly expressed in vessels, brain tissues, and blood [19,41,42,43]. Bioinformatic analysis revealed that HSPA8 also interacts with other proteins that control the regulation of protein constancy, protein folding, refolding, response to unfolded proteins, regulation of protein ubiquitination, and cellular response to unfolded proteins. The possible significance of HSPA8 (HSC70) in the molecular mechanisms of ischemic stroke has already been noted in previous studies. For example, according to Bustamente et al., elevated serum HSC70 levels are typical of ischemic stroke compared to hemorrhagic stroke [44]. Stankowski et al. noted that the heat shock C-terminus related to 70 interacting proteins increases after stroke and impairs survival in acute oxidative stress [45]. Previous studies also demonstrated that vascular expression of *HSPA8* had been up-regulated in individuals with atherosclerotic diseases, perhaps as a compensatory response [46,47]. HSPA8 was shown to interact with NLRC5 and suppress the NF-kB pathway [48] in macrophages, suggesting its role in vascular wall inflammation [49]. Noteworthy is that apparently HSPA8 may also display prothrombotic activities [50].

The functional impacts of genetic variants were interpreted using a bioinformatic approach because there has not been any research investigating the association of *HSPA8* SNPs with IS risk up to this point. This approach made it possible to study the molecular mechanisms behind the involvement of *HSPA8* polymorphic loci in IS pathogenesis.

Firstly, IS-linked SNPs are characterized by high regulatory potential: they significantly affect histone modifications in brain and blood tissues, mainly trimethylation in the 4th lysine residue of histone H3 and acetylation of lysine residues in the N-terminal position 27 of histone; they are largely regulated by regulatory proteins.

Secondly, according to bioinformatic resources, the risk alleles G rs10892958 and A rs1136141 have a relationship to cis-eQTL-mediated down-regulation of *HSPA8* in brain tissues. Allele A rs1136141 also affects an increase in *CLMP* expression in the brain. Probably, *CLMP* plays a substantial role in the formation of cerebral atherosclerosis since it encodes a type I transmembrane protein that is found in junctional complexes between endothelial and epithelial cells. This protein may also play a function in cell-cell adhesion [51].

Thirdly, risk allele G rs10892958 and risk allele A rs1136141 *HSPA8* produce DNA binding sites for TFs involved in oxidative stress, neurogenesis, angiogenesis, apoptosis, and migration of blood vessel endothelial cells; this adds more support to the notion that *HSPA8* plays a significant role in the molecular mechanisms underlying IS.

Fourth, information obtained with the bioinformatic tool CDKP revealed that the risk allele A rs1136141 is linked to a higher risk of cerebral stroke (TOAST other determined), increased level of systolic blood pressure, and an increased heart rate. As a result, this genetic variation may have a significant impact on the development of hypertension, the main risk factor for IS. Atrial fibrillation, another key risk factor for the cardioembolic type of IS, is also made more likely by a higher heart rate. Notably, CDKP data also indicates that the A allele rs1136141 has a role in risking peripheral artery disease in ever-smokers —a pathogenetically related cardiovascular pathology. This finding correlates with the data obtained in our study, which provide proof of the involvement of this genetic variant in the formation of IS risk in smokers.

On the other hand, HSPA8 may play a protective role in IS the process of ischemic stroke by protecting nerve cells and inhibiting neuronal apoptosis [52,53,54]. As a chaperone, HSPA8 orchestrates the folding and compartmentalization of the client proteins, contributing to the cellular stress response [55,56]. HSPA8 also down-regulates apoptotic cell death, both via direct suppression of several members of the apoptotic pathway and via activation of the anti-death protein bcl-2 [56]. Additionally, HSP70s have been shown to be critical for the biogenesis of extracellular vesicles, participating in neurological recovery after stroke [57]. Moreover, HSPA8 is considered a negative regulator of oxidative stress. Some authors revealed that constitutively expressed HSPA8 plays a role in protecting cardiomyocytes from oxidative damage, increasing cell survival [58,59]. Finally, *Hspa1a/ Hspa1b* knock-out mice subjected to different thrombotic challenges developed thrombosis significantly earlier than their wild-type counterparts, suggesting the important role of members of HSP70 family in regulation of haemostasis [60]. More recent studies have reported that HSPA8 is released from cardiomyocytes under oxidative stress [61]. Altogether, these data indicate that HSPA8 may take an important place during the cellular response to ischemia, and future research should address its role in the course of IS.

In our study, we also revealed sex-specific correlates of *HSPA8* genetic variants. Numerous proofs are presented in the literature that candidate genes for cardiovascular diseases are characterized by pronounced gender-specific effects in the manifestation of associations [62,63,64,65,66]. The association of rs10892958 with the development of IS in men found in our study is explained by the regulation of *HSPA8* expression by sex hormones. There is a lot of evidence that estradiol increases the expression of *HSPA8* [67,68,69,70,71]. Considering that the carriage of the G rs10892958 allele is associated with *HSPA8* down-regulation, the absence of a compensating effect of estrogens on the *HSPA8* mRNA level increases the risk effects of this genetic variant. In addition, it has been found that in women, *HSPA8* levels are inversely correlated with the level of Toll-like receptor 4, which triggers oxidative stress and inflammation, which are key elements of vascular diseases [72]. At the same time, no such correlation has been found in men.

In the present research, we also report environmentally-dependent effects of *HSPA8* polymorphisms. It is known that environmental risk factors can modify the contribution of genetic variants on the risk of diseases [28,73,74]. Our study provided significant evidence of the role of smoking and low consumption of fresh vegetables and fruits in the manifestation of associations rs10892958 and rs1136141 *HSPA8* and the development of IS. It is noteworthy that both of these risk factors are associated with high levels of reactive oxygen species [75,76] and, accordingly, oxidative stress, a major link contributing to the development of cardiovascular disorders [25,77,78,79]. Smoking-specific correlates of candidate genes with risk of IS and stroke-related phenotypes have been demonstrated in many of our prior investigations [65,80], suggesting a significant role for smoking-induced endothelial dysfunction, vascular tone disorders, and atherosclerosis in the development of IS. The Comparative Toxigenomics Database offered proof of smoking’s effects on the reduction of *HSPA8* expression [81] as well as accelerating the metabolism of HSPA8 protein [82]. Thus, most likely, smoking exacerbates the effect of IS-related alleles on the risk of disease by down-regulating *HSPA8* expression.

In addition to smoking, low fruit and vegetable intake is linked to high levels of reactive oxygen species, in particular hydroperoxides [83,84], and increased consumption of antioxidant-rich foods may have prompted a decrease in the total amount of lipid peroxide [85]. In vitro research demonstrates that polyphenols, which are rich in fresh vegetables and fruits, shield arteries and neural cells from the toxicity of oxidative glutamate and H_2_O_2_ [86,87]. Many polyphenols have strong anti-inflammatory activities in addition to their anti-oxidant characteristics [88]. The conducted studies have shown that hydrogen peroxide may affect the level of *HSPA8* mRNA [89], resulting in decreased expression of the HSPA8 protein [90]. Thus, low fresh fruit and vegetable consumption related to oxidative stress and high levels of reactive oxygen species may affect the decrease in *HSPA8* expression, thereby showing more pronounced effects of the risk alleles rs10892958 and rs1136141 *HSPA8*.

In conclusion, the results of our case-control study indicate that susceptibility to IS may be determined by genetic variants in *HSPA8*. However, we examined only tagSNPs and excluded from the analysis SNPs that are in linkage disequilibrium with tagSNPs, which can lead to false-positive results since the relationships we identified may reflect the effects of genetic variants linked to analysed SNPs. This is a limitation of our study. Further research is needed to confirm our findings and explore potential molecular mechanisms underlying the associations between IS risk and polymorphisms in the *HSPA8* gene.

## Figures and Tables

**Table 1:** Baseline and clinical characteristics of the studied groups.

Baseline and Clinical Characteristics		IS Patients (N = 888)	Controls (N = 1251)	*p*-Value
Age, Me [Q1; Q3]		62 [55; 69]	58 [53; 66]	**<0.001**
Gender	Males, N (%)	481 (54.2%)	577 (46.1%)	**<0.001**
	Females, N (%)	407 (45.8%)	674 (53.9%)	
Smoking	Yes, N (%)	425 (47.9%)	331 (26.5%)	**<0.001**
	No, N (%)	463 (52.1%)	920 (73.5%)	
Hypodynamia	Yes, N (%)	332 (39.34%)	ND	
	No, N (%)	512 (60.66%)		
Low fruit/vegetable consumption	Yes, N (%)	449 (53.20%)	ND	
	No, N (%)	395 (46.80%)		
Type 2 diabetes mellitus	Yes, N (%)	103 (11,6%)	-	
	No, N (%)	740 (83,3%)	-	
	ND, N (%)	45 (5,1%)	-	
Body mass index, Me [Q1; Q3]		23 [22; 26] (N = 567)	-	
Family history of cerebrovascular diseases	Yes, N (%)	296 (35.20%)	ND	
	No, N (%)	545 (64.80%)	ND	
Age at onset of stroke, Me [Q1; Q3]		61 [54; 69] (N = 862)	-	
Number of strokes including event in question	1, N (%)	766 (88.86%)	-	
	2, N (%)	85 (9.86%)	-	
	3, N (%)	11 (1.28%)	-	
Stroke localization	Right/left middle cerebral artery basin, N (%)	720 (83.82%)	-	
	Vertebrobasilar basin, N (%)	139 (16.18%)	-	
Area of lesion in stroke, mm^2^, Me [Q1; Q3]		105.00 [28; 468] (N = 841)	-	
Total cholesterol, mmol/L, Me [Q1; Q3]		5.2 [4.4; 5.8] (N = 583)	ND	
Triglycerides, mmol/L, Me [Q1; Q3]		1.3 [1.1; 1.8] (N = 577)	ND	
Glucose level, mmol/L, Me [Q1; Q3]		4.7 [4.3; 5.5] (N = 849)	ND	
Prothrombin time, seconds, Me [Q1; Q3]		10.79 [10.14; 11.70] (N = 839)	ND	
International normalized ratio, Me [Q1; Q3]		1 [0.94; 1.09] (N = 573)	ND	
Activated partial thromboplastin time, seconds, Me [Q1; Q3]		32.7 [29; 37] (N = 576)	ND	

Statistically significant differences between groups are indicated in bold; “-”— the characteristic is absent in healthy individuals; ND—no data.

**Table 2:** Analysis of relationships between *HSPA8* SNPs IS risk.

Genetic Variant	Effect Allele	Other Allele	N	OR [95% CI] ^1^	*p*^2^ (P_bonf_)
Entire group					
rs1461496	A	G	2132	1.00 [0.88–1.15]	0.95
rs10892958	G	G	2138	1.16 [0.99–1.35]	0.06
rs1136141	A	G	2024	1.09 [0.90–1.30]	0.38
Males					
rs1461496	A	G	1065	1.01 [0.85; 1.21]	0.9
rs10892958	G	G	1057	**1.30 [1.05; 1.61]**	**0.01**
rs1136141	A	G	999	1.08 [0.84; 1.40]	0.55
Females					
rs1461496	A	G	1076	1.05 [0.87; 1.26]	0.63
rs10892958	G	G	1081	1.08 [0.87; 1.33]	0.49
rs1136141	A	G	1025	1.16 [0.91; 1.47]	0.24
Nonsmokers (f−)					
rs1461496	A	G	1379	1.10 [0.93; 1.31]	0.24
rs10892958	G	G	1383	1.07 [0.88; 1.30]	0.51
rs1136141	A	G	1306	0.87 [0.68; 1.10]	0.23
Smokers (f+)					
rs1461496	A	G	753	0.86 [0.70; 1.07]	0.18
rs10892958	G	G	755	**1.37 [1.07; 1.77]**	**0.01**
rs1136141	A	G	718	**1.68 [1.23; 2.28]**	**7.0 × 10^−4^**
Normal fruit and vegetable intake (f−)					
rs1461496	A	G	1639	1.02 [0.86; 1.20]	0.86 (_bonf_1.0)
rs10892958	G	G	1645	1.03 [0.84; 1.25]	0.78 (_bonf_1.0)
rs1136141	A	G	1559	0.91 [0.72; 1.16]	0.45 (_bonf_ 0.9)
Low fruit and vegetable intake (f+)					
rs1461496	A	G	1694	1.07 [0.91; 1.25]	0.42 (_bonf_0.84)
rs10892958	G	G	1699	**1.36 [1.14; 1.63]**	**9.0 × 10^−4^ (_bonf_0.002)**
rs1136141	A	G	1608	**1.29 [1.05; 1.60]**	**0.02 (_bonf_0.04)**

^1^—odds ratio and 95% confidence interval; ^2^—*p*-level (bonf—*p*-level with Bonferroni correction). All the calculations were done considering the minor alleles (effect alleles). Bold was used to indicate statistically significant differences.

**Table 3:** Cis-eQTL-mediated effects of *HSPA8* SNPs (http://www.mulinlab.org/qtlbase (accessed on 21 February 2023)).

SNP	Trait	Effect Allele	Tissue	Effect Size (Beta)	PVAL	FDR
rs10892958	*HSPA8*	G	Brain-Hippocampus	−0.44	1.9 × 10^−7^	5.8 × 10^−5^
rs1136141	*HSPA8*	A	Brain-Hippocampus	−0.12	3.8 × 10^−5^	0.006
	*CLMP*	A	Brain-Hippocampus	0.13	4.8 × 10^−5^	0.008

**Table 4:** The impact of HSPA8 SNPs on histone tags in various tissues.

SNP (Ref/Alt Allele)	Tissues Marks	Brain							Blood
		(1)	(2)	(3)	(4)	(5)	(6)	(7)	(8)
rs10892958 (C/**G**)	H3K4me1	No	No	No	E	No	No	E	E
	H3K4me3	P	P	P	P	P	P	P	P
	H3K27ac	E	E	E	E	E	E	E	E
	H3K9ac	No	P	P	P	P	P	P	P
	DNase	No	No	No	No	No	No	No	DNase
rs1136141 (G/**A**)	H3K4me1	No	No	No	No	No	No	No	E
	H3K4me3	P	P	P	P	P	P	P	P
	H3K27ac	E	E	E	E	E	E	E	E
	H3K9ac	No	P	P	P	P	P	P	P
	DNase	No	No	No	No	No	No	No	DNase

H3K4me1—histone H3 lysine 4 mono-methylation; H3K4me3—histone H3 lysine 4 tri-methylation; H3K9ac—the acetylation at the 9th lysine residues of the histone H3 protein; H3K27ac—acetylation of lysine 27 on histone H3 protein subunit; effect alleles are marked in bold. E—histone modification in the enhancer region; P—histone modification at the promoter region. 1—brain hippocampus middle; 2—brain substantia nigra; 3—brain anterior caudate; 4—brain cingulate gyrus; 5—brain inferior temporal lobe; 6—brain angular gyrus; 7—brain dorsolateral prefrontal cortex; 8—Cells from peripheral blood (any); No—No histone modifications; Effect alleles are marked in bold.

**Table 5:** Results of aggregated analyses of associations between *HSPA8* SNPs and cerebrovascular diseases/ their intermediate phenotypes (CDKP: Cerebrovascular Disease Knowledge Portal data).

No.	SNP	Phenotype	*p*-Value	Beta (OR)	Sample Size
1.	rs1136141 (G/**A**)	Systolic blood pressure	0.008	_Beta_▲0.0056	1,325,890
2.		Heart rate	0.01	_Beta_▲0.007	484,178
3.		Peripheral artery disease in ever-smokers	0.04	_OR_▲1.0639	28,235
4.		TOAST, other-determined	0.03	_OR_▲2.4103	9277

Effect allele is marked in bold.

## Data Availability

The data presented in this study are available upon request from the corresponding author.

## Supplementary Materials

The following supporting information can be downloaded at https://www.mdpi.com/article/10.3390/genes14061171/s1. Figure S1: Plot showing a clear separation between the signals derived from allele 1 (rs1136141-G, FAM fluorescent dye) or allele 2 (rs1136141-A, ROX fluorescent dye). Genotypes GG, GA, and AA are shown as circles, triangles, and squares, respectively; Figure S2: Plot showing a clear separation between the signals derived from allele 1 (rs1461496-A, FAM fluorescent dye) or allele 2 (rs1461496-G, ROX fluorescent dye). Genotypes GG, GA, and AA are shown as squares, triangles, and circles, respectively; Figure S3: Plot showing a clear separation between the signals derived from allele 1 (rs1136141-C, FAM fluorescent dye) or allele 2 (rs1136141-G, ROX fluorescent dye). Genotypes CC, CG, and GG are shown as circles, triangles, and squares, respectively; Table S1: Primers and probes designed for this study; Table S2: Main functional characteristics of predicted functional partners of *HSPA8*; Table S3: Functional enrichments of HSPA8 network; Table S4: Distribution of *HSPA8* genotypes in ischemic stroke patients/healthy controls and their correspondence to the Hardy–Weinberg equilibrium; Table S5: Distribution of *HSPA8* genotypes in sub-group analysis of patients/healthy controls; Table S6: Analysis of the effect of rs10892958 of *HSPA8* on binding of DNA to transcription factors; Table S7: Analysis of the effect of rs1136141 of *HSPA8* on binding of DNA to transcription factors.
