# Supplementary material for: Association between HSPA8 Gene Variants and Ischemic Stroke: A Pilot Study Providing Additional Evidence for the Role of Heat Shock Proteins in Disease Pathogenesis
Source: Genes (Basel). 2023 May 27;14(6):1171. doi: 10.3390/genes14061171 (PMC10298525; doi:10.3390/genes14061171)
Supplement: Supplementary file 1 [file genes-14-01171-s001.zip › 25.05.2023 supplementary materials.pdf]

Table S1: Primers and probes were designed for this study

rs1136141 *HSPA8*	5′- AGAACCCCCAAGCCTCAG -3′<br>5′- GGAAACCGGTCTCATTGAACT -3′<br>5′- FAM-TACCTGGG**G**TGTAGGCC-RTQ1-3′<br>5′- ROX-TACCTGGG**A**TGTAGGCC-BHQ2-3′
rs1461496 *HSPA8*	5′- CTCGCCTTCATAAACCTTGG -3′<br>5′- TGGATTCCAAAACCATTCGT -3′<br>5′- FAM-TCGCTCAAACATCCA-RTQ1-3′<br>5′- ROX-TCGCTCAGACATCCA-BHQ2-3′
rs10892958 *HSPA8*	5′- CCTTGGACATGGTTGCTGAA -3′<br>5′- CGTGCTTAGACAGGAGGTGA-3′<br>5′- FAM-CCAGGAAAAACGTATGGCCACTGC-RTQ1-3′<br>5′- ROX-CCAGGAAAAAGGTATGGCCACTGC-BHQ2-3′

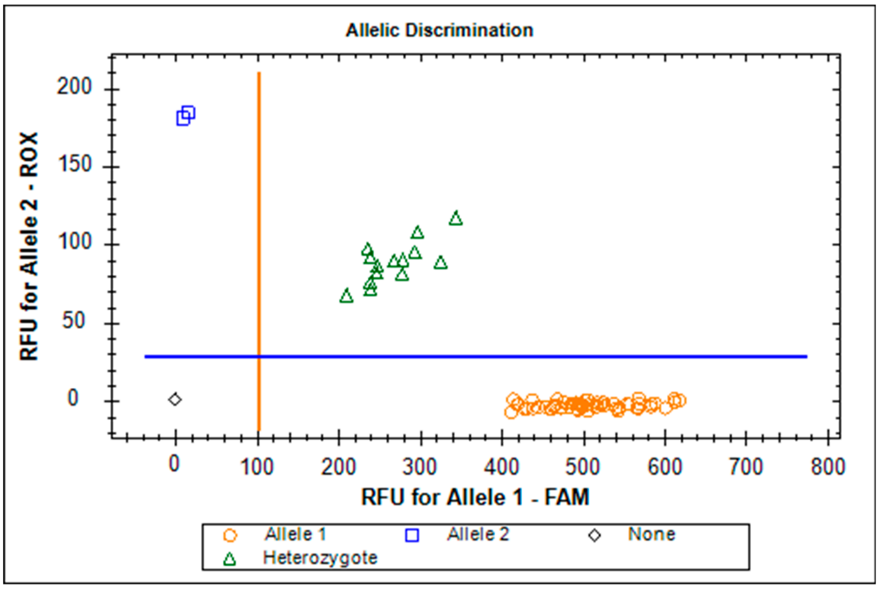

Figure S1: The plot shows clear separation between the signals derived from allele 1 (rs1136141-G, FAM fluorescent dye) or allele 2 (rs1136141-A, ROX fluorescent dye). Genotypes GG, GA, and AA are shown as circles, triangles, and squares, respectively

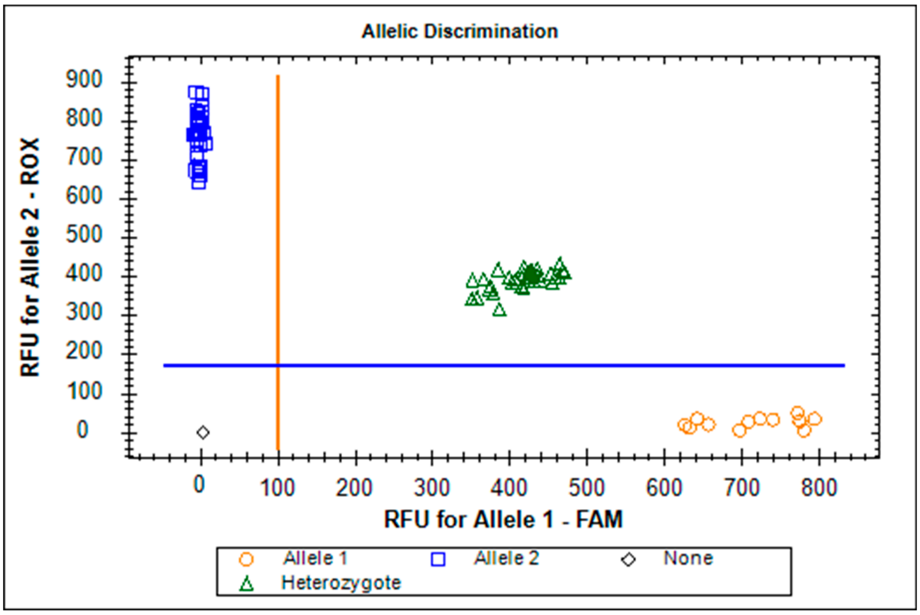

Figure S2: The plot shows clear separation between the signals derived from allele 1 (rs1461496-A, FAM fluorescent dye) or allele 2 (rs1461496-G, ROX fluorescent dye). Genotypes GG, GA, and AA are shown as squares, triangles, and circles, respectively.

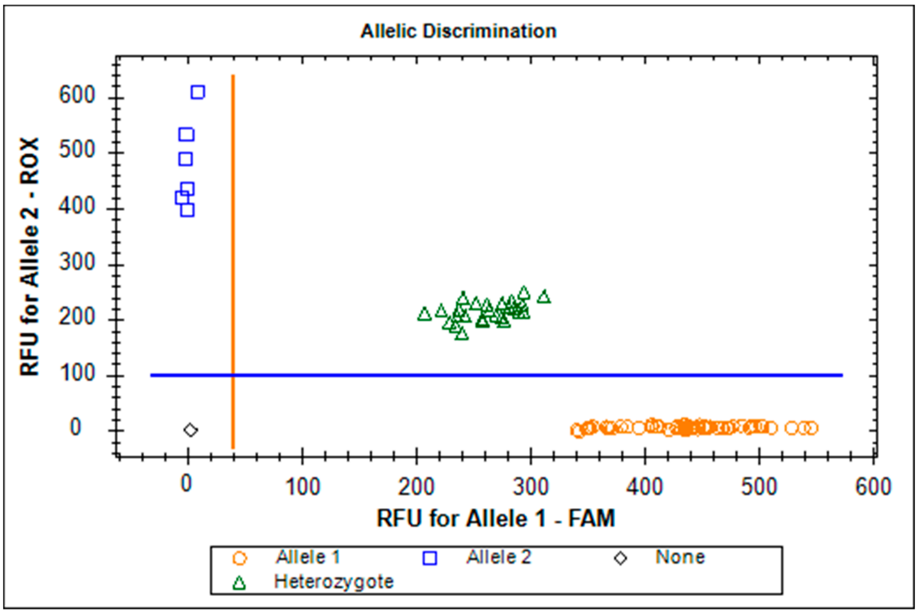

Figure S3: The plot shows clear separation between the signals derived from allele 1 (rs1136141-C, FAM fluorescent dye) or allele 2 (rs1136141-G, ROX fluorescent dye). Genotypes CC, CG, and GG are shown as circles, triangles, and squares, respectively.

Table S2: Main functional characteristics of predicted functional partners of HSPA8 (only experimentally confirmed data)

Protein	Functions	Score
STIP1	Stress-induced-phosphoprotein 1; Acts as a co-chaperone for HSP90AA1. Mediates the association of the molecular chaperones HSPA8/HSC70 and HSP90.	0.989
BAG1	Co-chaperone for HSP70 and HSC70 chaperone proteins. Acts as a nucleotide-exchange factor (NEF) promoting the release of ADP from the HSP70 and HSC70 proteins thereby triggering client/substrate protein release. Inhibits the pro-apoptotic function of PPP1R15A, and has anti-apoptotic activity. Markedly increases the anti-cell death function of BCL2 induced by various stimuli.	0.988
BAG2	Co-chaperone for HSP70 and HSC70 chaperone proteins. Acts as a nucleotide-exchange factor (NEF) promoting the release of ADP from the HSP70 and HSC70 proteins thereby triggering client/substrate protein release.	0.954
STUB1	E3 ubiquitin-protein ligase which targets misfolded chaperone substrates towards proteasomal degradation. Collaborates with ATXN3 in the degradation of misfolded chaperone substrates. Modulates the activity of several chaperone complexes, including Hsp70, Hsc70 and Hsp90. Acts as a co-chaperone for HSPA1A and HSPA1B chaperone proteins and promotes ubiquitin-mediated protein degradation. Negatively regulates the suppressive function of regulatory T-cells (Treg) during inflammation.	0.942

	Negatively regulates TGF-beta signaling. May regulate myosin assembly in striated muscles together with UBE4B and VCP/p97.	
HSP90AA1	Molecular chaperone that promotes the maturation, structural maintenance and proper regulation of specific target proteins involved for instance in cell cycle control and signal transduction. Undergoes a functional cycle that is linked to its ATPase activity which is essential for its chaperone activity. Engages with a range of client protein classes via its interaction with various co-chaperone proteins or complexes, that act as adapters, simultaneously able to interact with the specific client and the central chaperone itself. Plays a critical role in mitochondrial import, delivers preproteins to the mitochondrial import receptor TOMM70. Apart from its chaperone activity, it also plays a role in the regulation of the transcription machinery. HSP90 and its co-chaperones modulate transcription. Antagonizes STUB1-mediated inhibition of TGF-beta signaling via inhibition of STUB1-mediated SMAD3 ubiquitination and degradation.	0.936
BAG3	Co-chaperone for HSP70 and HSC70 chaperone proteins. Acts as a nucleotide-exchange factor (NEF) promoting the release of ADP from the HSP70 and HSC70 proteins thereby triggering client/substrate protein release. Has anti-apoptotic activity. Plays a role in the HSF1 nucleocytoplasmic transport.	0.915
HSPA4	Predicted to enable ATP binding activity. Involved in chaperone-mediated protein complex assembly and protein insertion into mitochondrial outer membrane. Located in cytosol and extracellular exosome.	0.911
HSPBP1	Inhibits HSPA1A chaperone activity by changing the conformation of the ATP-binding domain of HSPA1A and interfering with ATP binding. Interferes with ubiquitination mediated by STUB1 and inhibits chaperone-assisted degradation of immature CFTR.	0.898
LRRK2	Serine/threonine-protein kinase which phosphorylates a broad range of proteins involved in multiple processes such as neuronal plasticity, autophagy, and vesicle trafficking. Is a key regulator of RAB GTPases by regulating the GTP/GDP exchange and interaction partners of RABs through phosphorylation. Regulates neuronal process morphology in the intact central nervous system (CNS). Plays a role in synaptic vesicle trafficking. Positively regulates autophagy through a calcium-dependent activation of the CaMKK/AMPK signaling pathway.	0.888
DNAJA1	Co-chaperone for HSPA8/Hsc70. Plays a role in protein transport into mitochondria via its role as co-chaperone. Functions as co-chaperone for HSPA1B and negatively regulates the translocation of BAX from the cytosol to mitochondria in response to cellular stress, thereby protecting cells against apoptosis.	0.885
Data from the STRING database is shown.<br>* - Proven interaction mechanisms based only on experiments.		

Table S3: Functional enrichments of HSPA8 network (only experimentally confirmed data)

№	Term ID	Term Description	Observed Gene Count	Background Gene Count	Strength	FDR
	Biological processes (Gene Ontology)					
1	GO:0006457	Protein folding	7	213	1.77	8.40×10^{-8}
2	GO:0061684	Chaperone-mediated autophagy	4	8	2.95	1.71×10^{-7}
3	GO:0006986	Response to unfolded protein	6	166	1.81	8.04×10^{-7}

4	GO:1900034	Regulation of cellular response to heat	6	79	2.13	1.50×10^{-6}
5	GO:0031396	Regulation of protein ubiquitination	4	212	1.53	1.68×10^{-6}
6	GO:0031647	Regulation of protein stability	5	293	1.48	8.40×10^{-6}
7	GO:1903364	Positive regulation of cellular protein catabolic process	5	161	1.74	2.61×10^{-5}
8	GO:1903748	Negative regulation of establishment of protein localization to mitochondrion	3	9	2.77	3.72×10^{-5}
9	GO:0031331	Positive regulation of cellular catabolic process	6	397	1.43	3.72×10^{-5}
10	GO:0080135	Regulation of cellular response to stress	7	739	1.23	3.72×10^{-5}
11	GO:0070585	Protein localization to mitochondrion	4	81	1.94	9.51×10^{-5}
12	GO:0031329	Regulation of cellular catabolic process	5	875	1.01	9.51×10^{-5}
13	GO:0009408	Response to heat	4	101	1.85	1.70×10^{-4}
14	GO:0006914	Autophagy	6	270	1.52	1.70×10^{-4}
15	GO:1901800	Positive regulation of proteasomal protein catabolic process	4	122	1.77	3.00×10^{-4}
16	GO:0045040	Protein insertion into mitochondrial outer membrane	2	4	2.95	0.002
17	GO:0034605	Cellular response to heat	3	60	1.95	0.002
18	GO:0050790	Regulation of catalytic activity	7	2386	0.72	0.002
19	GO:1903215	Negative regulation of protein targeting to mitochondrion	2	7	2.71	0.004
20	GO:0072321	Chaperone-mediated protein transport	2	11	2.51	0.008
21	GO:0032436	Positive regulation of proteasomal ubiquitin-dependent protein catabolic process	3	100	1.73	0.008
22	GO:0032091	Negative regulation of protein binding	3	106	1.7	0.009
23	GO:0033365	Protein localization to organelle	5	743	1.08	0.010
24	GO:0010033	Response to organic substance	8	3011	0.67	0.011
25	GO:0031398	Positive regulation of protein ubiquitination	3	126	1.63	0.013
26	GO:0034620	Cellular response to unfolded protein	3	129	1.62	0.014
27	GO:0042026	Protein refolding	2	18	2.3	0.016
28	GO:0017038	Protein import	3	144	1.57	0.018
29	GO:0051131	Chaperone-mediated protein complex assembly	2	21	2.23	0.018
30	GO:0072594	Establishment of protein localization to organelle	4	433	1.22	0.018
31	GO:0032880	Regulation of protein localization	5	934	0.98	0.022
32	GO:0034613	Cellular protein localization	6	1610	0.82	0.023
33	GO:0006886	Intracellular protein transport	5	999	0.95	0.027
34	GO:0051085	Chaperone cofactor-dependent protein refolding	2	29	2.09	0.028
35	GO:0050821	Protein stabilization	3	186	1.46	0.029
36	GO:0051338	Regulation of transferase activity	5	1036	0.93	0.030
37	GO:0038128	ERBB2 signaling pathway	2	32	2.05	0.032
38	GO:1903827	Regulation of cellular protein localization	4	568	1.1	0.039
39	GO:0044092	Negative regulation of molecular function	5	1163	0.88	0.046
40	GO:0051223	Regulation of protein transport	4	617	1.06	0.049
Data from the STRING database is shown.						

Table S4: Distribution of *HSPA8* genotypes in ischemic stroke patients/healthy controls and their correspondence to the Hardy-Weinberg equilibrium

SNPs	Genotypes	Controls	H_o (H_e)[1]	P[2]	IS patients	H_o (H_e)[3]	P[4]
rs1461496	G/G	496 (39.8%)	0.4691 (0.4647)	>0.05	335 (37.8%)	0.4972 (0.4681)	>0.05
	G/A	584 (46.9%)			441 (49.7%)		

	A/A	165 (13.2%)			111 (12.5%)		
rs10892958	C/C	803 (64.2%)	0.3144 (0.3205)	>0.05	521 (58.7%)	0.3694 (0.3527)	>0.05
	C/G	393 (31.4%)			328 (36.9%)		
	G/G	54 (4.3%)			39 (4.4%)		
rs1136141	G/G	893 (75.5%)	0.2234 (0.2304)	>0.05	623 (74%)	0.2280 (0.2495)	<0.05
	A/G	264 (22.3%)			192 (22.8%)		
	A/A	25 (2.1%)			27 (3.2%)		
[1] – observed (Ho) and expected (He) heterozygosity in healthy controls; [2] – P-HWE in healthy controls; [3] – observed (Ho) and expected (He) heterozygosity in IS patients; [4] – P-HWE in IS patients							

Table S5: Distribution of *HSPA8* genotypes in subgroups analysis of patients/healthy controls

SNP	Genotypes	Controls	IS patients	Controls	IS patients
	Males (controls (N=577)/IS patients (N=481))			Females (controls (N=674)/IS patients (N=407))	
rs1461496	G/G	232 (40.4%)	178 (37%)	264 (39.4%)	157 (38.7%)
	G/A	260 (45.2%)	247 (51.4%)	324 (48.4%)	194 (47.8%)
	A/A	83 (14.4%)	56 (11.6%)	82 (12.2%)	55 (13.6%)
	Maf (A)	0.370	0.373	0.364	0.374
rs10892958	C/C	375 (65.1%)	276 (57.4%)	428 (63.5%)	245 (60.2%)
	C/G	179 (31.1%)	182 (37.8%)	214 (31.8%)	146 (35.9%)
	G/G	22 (3.8%)	23 (4.8%)	32 (4.8%)	16 (3.9%)
	Maf (G)	0.194	0.237	0.206	0.219
rs1136141	G/G	415 (75.9%)	341 (75.4%)	478 (75.3%)	282 (72.3%)
	A/G	124 (22.7%)	98 (21.7%)	140 (22.1%)	94 (24.1%)
	A/A	8 (1.5%)	13 (2.9%)	17 (2.7%)	14 (3.6%)
	Maf (A)	0.128	0.137	0.137	0.156
	Nonsmokers (f-) (controls (N=920)/IS patients (N=)463)			Smokers (f+) (controls (N=331)/IS patients (N=425))	
rs1461496	G/G	378 (41.2%)	174 (37.7%)	118 (36%)	161 (37.9%)
	G/A	430 (46.9%)	229 (49.6%)	154 (47%)	212 (49.9%)
	A/A	109 (11.9%)	59 (12.8%)	56 (17.1%)	52 (12.2%)
	Maf (A)	0.353	0.376	0.405	0.372
rs10892958	C/C	583 (63.4%)	280 (60.5%)	220 (66.7%)	241 (56.7%)
	C/G	298 (32.4%)	167 (36.1%)	95 (28.8%)	161 (37.9%)
	G/G	39 (4.2%)	16 (3.5%)	15 (4.5%)	23 (5.4%)
	Maf (G)	0.204	0.215	0.189	0.244
rs1136141	G/G	640 (73.8%)	336 (76.5%)	253 (80.3%)	287 (71.2%)
	A/G	205 (23.6%)	95 (21.6%)	59 (18.7%)	97 (24.1%)
	A/A	22 (2.5%)	8 (1.8%)	3 (1%)	19 (4.7%)
	Maf (A)	0.144	0.126	0.103	0.167
	Normal fruit and vegetable intake (f-) (controls (N=1251)/IS patients (N=395))			Low fruit and vegetable intake (f+) (controls (N=1251)/IS patients (N=449))	
rs1461496	G/G	496 (39.8%)	152 (38.6%)	496 (39.8%)	163 (36.3%)
	G/A	584 (46.9%)	192 (48.7%)	584 (46.9%)	229 (51%)
	A/A	165 (13.2%)	50 (12.7%)	165 (13.2%)	57 (12.7%)
	Maf (A)	0.367	0.371	0.367	0.382
rs10892958	C/C	803 (64.2%)	250 (63.3%)	803 (64.2%)	243 (54.1%)
	C/G	393 (31.4%)	128 (32.4%)	393 (31.4%)	184 (41%)

	G/G	54 (4.3%)	17 (4.3%)	54 (4-.3%)	22 (4.9%)
	Maf (G)	0.200	0.205	0.200	0.254
rs1136141	G/G	893 (75.5%)	296 (78.5%)	893 (75.5%)	300 (70.4%)
	A/G	264 (22.3%)	70 (18.6%)	264 (22.3%)	110 (25.8%)
	A/A	25 (2.1%)	11 (2.9%)	25 (2.1%)	16 (3.8%)
	Maf (A)	0.133	0.122	0.133	0.167

Table S6: Analysis of the effect of rs10892958 *HSPA8* on binding of DNA to transcription factors

№	Ref/ SNP allele[1]	TF[2]	GAIN /LOSS[3]	Motif[4]	P-Value SNP impact[5]	P-Value Ref[6]	P-Value SNP[7]
1	C/G	AP1	gain	AP1_disc9	0	0.040	0.361
2	C/G	OLIG1	gain	OLIG1_1	0.001	0.001	0.200
3	C/G	CDC5L	gain	CDC5L_1	0.001	0.010	0.239
4	C/G	NEUROG2	gain	NEUROG2_2	0.002	0.002	0.112
5	C/G	OLIG3	gain	OLIG3_1	0.002	0.001	0.065
6	C/G	FOXO4	gain	FOXO4_3	0.003	0.012	0.482
7	C/G	OLIG2	gain	OLIG2_1	0.003	0.001	0.056
8	C/G	FOXD3	gain	FOXD3_2	0.005	0.009	0.099
9	C/G	CENPB	gain	CENPB_1	0.006	0.015	0.369
10	C/G	FOXA1	gain	FOXA1_1	0.006	0.033	0.553
11	C/G	HES5	gain	HES5_1	0.007	0.021	0.279
12	C/G	RUNX3	gain	RUNX3_3	0.009	0.013	0.159
13	C/G	ARNT	gain	ARNT_3	0.010	0.024	0.386
14	C/G	USF1	gain	USF1_1	0.010	0.025	0.273
15	C/G	USF	gain	USF_1	0.011	0.039	0.396
16	C/G	HDAC2	gain	HDAC2_disc2	0.012	0.019	0.257
17	C/G	FOXD2	gain	FOXD2_1	0.015	0.041	0.372
18	C/G	BARHL2	gain	BARHL2_2	0.015	0.017	0.280
19	C/G	HLF	gain	HLF_4	0.016	0.026	0.342
20	C/G	MYC::MAX	gain	MYC::MAX_2	0.017	0.047	0.460
21	C/G	ERG	gain	AP1_disc9	0.000	0.040	0.361
22	C/G	IRF	gain	OLIG1_1	0.001	0.001	0.200
23	C/G	IRF2	gain	CDC5L_1	0.001	0.010	0.239
24	C/G	MYC::MAX	gain	NEUROG2_2	0.002	0.002	0.112
25	C/G	HOMEZ	gain	OLIG3_1	0.002	0.001	0.065
26	C/G	FOXO6	gain	FOXO4_3	0.003	0.012	0.482
27	C/G	SP2	gain	OLIG2_1	0.003	0.001	0.056
28	C/G	SIX3	gain	FOXD3_2	0.005	0.009	0.099
29	C/G	E4F1	gain	CENPB_1	0.006	0.015	0.369
30	C/G	MYBL2	gain	FOXA1_1	0.006	0.033	0.553
31	C/G	FOXD2	gain	HES5_1	0.007	0.021	0.279
32	C/G	SPI1	loss	SPI1_disc3	0	0.200	0
33	C/G	EN1	loss	EN1_2	0	0.675	0
34	C/G	PPARG	loss	PPARG_4	3.8×10^{-4}	0.474	0.011
35	C/G	PPARG::RXRA	loss	MA0065.2	0.001	0.426	0.011
36	C/G	IRF	loss	IRF_disc4	0.002	0.426	0.015
37	C/G	BDP1	loss	BDP1_disc3	0.002	0.316	0.031
38	C/G	REL	loss	REL_2	0.003	0.424	0.015

39	C/G	ZNF784	loss	ZNF784_1	0.003	0.624	0.015
40	C/G	ZEB1	loss	ZEB1_disc1	0.003	0.237	0.011
41	C/G	IRF1	loss	IRF1_2	0.003	0.277	0.017
42	C/G	Sox3	loss	MA0514.1	0.003	0.186	0.012
43	C/G	NFIC	loss	NFIC_1	0.004	0.170	0.027
44	C/G	TEAD1	loss	TEAD1_3	0.004	0.527	0.013
45	C/G	TEAD1	loss	TEAD1_1	0.005	0.286	0.011
46	C/G	KLF4	loss	KLF4_1	0.005	0.482	0.046
47	C/G	TATA	loss	TATA_disc4	0.006	0.099	0.006
48	C/G	SOX2	loss	SOX2_1	0.006	0.079	0.006
49	C/G	ESRRA	loss	ESRRA_6	0.006	0.302	0.044
50	C/G	EP300	loss	EP300_disc10	0.007	0.126	0.011
51	C/G	CACD	loss	CACD_1	0.007	0.278	0.046
52	C/G	IRF8	loss	IRF8_3	0.008	0.476	0.033
53	C/G	Sox6	loss	MA0515.1	0.008	0.165	0.012
54	C/G	KLF7	loss	KLF7_1	0.008	0.421	0.048
55	C/G	Klf4	loss	MA0039.2	0.009	0.453	0.049
56	C/G	Sox2	loss	MA0143.3	0.011	0.187	0.025
57	C/G	RARG	loss	RARG_5	0.011	0.526	0.047
58	C/G	HLTF	loss	HLTF_1	0.014	0.190	0.013
59	C/G	En1	loss	MA0027.1	0.014	0.386	0.022
60	C/G	YY1	loss	YY1_disc1	0.014	0.326	0.034
61	C/G	Klf1	loss	MA0493.1	0.016	0.339	0.032
62	C/G	MYC::MAX	loss	SPI1_disc3	0	0.200	0
63	C/G	NANOG	loss	EN1_2	0	0.675	0
64	C/G	PRDM1	loss	PPARG_4	$3.8{\times}10^{-4}$	0.474	0.011
65	C/G	AP1	loss	MA0065.2	0.001	0.426	0.011
66	C/G	Foxd3	loss	IRF_disc4	0.002	0.426	0.015
67	C/G	FOXD3	loss	BDP1_disc3	0.002	0.316	0.031
68	C/G	IKZF3	loss	REL_2	0.003	0.424	0.015
69	C/G	TAL1::TCF3	loss	ZNF784_1	0.003	0.624	0.015
70	C/G	GMEB2	loss	ZEB1_disc1	0.003	0.237	0.011
71	C/G	CLOCK	loss	IRF1_2	0.003	0.277	0.017
72	C/G	MYBL1	loss	MA0514.1	0.003	0.186	0.012
73	C/G	CREB3L1	loss	NFIC_1	0.004	0.170	0.027
74	C/G	MYBL2	loss	TEAD1_3	0.004	0.527	0.013
75	C/G	MYB	loss	TEAD1_1	0.005	0.286	0.011
76	C/G	FLI1	loss	KLF4_1	0.005	0.482	0.046
77	C/G	IRF7	loss	TATA_disc4	0.006	0.099	0.006
78	C/G	NFKB	loss	SOX2_1	0.006	0.079	0.006
79	C/G	SNAI2	loss	ESRRA_6	0.006	0.302	0.044
80	C/G	IRF1	loss	EP300_disc10	0.007	0.126	0.011
81	C/G	SCRT1	loss	CACD_1	0.007	0.278	0.046
82	C/G	SOX8	loss	IRF8_3	0.008	0.476	0.033
83	C/G	HNF4	loss	MA0515.1	0.008	0.165	0.012
84	C/G	GMEB2	loss	KLF7_1	0.008	0.421	0.048
	G [8]	regulation of transcription from RNA polymerase II promoter in response to oxidative stress (GO:0043619; FDR=0.02);<br>glial cell fate commitment (GO:0021781; FDR=0.04);<br>regulation of oligodendrocyte differentiation (GO:0048713; FDR=0.01);<br>neuron fate commitment (GO:0048663; FDR=0.0009);<br>negative regulation of neuron differentiation (GO:0045665: FDR=0.04);					

		oligodendrocyte differentiation (GO:0048709; FDR=0.04)
	C[9]	macrophage derived foam cell differentiation (GO:0010742; FDR=0.0149);<br>glial cell fate commitment (GO:0021781; FDR=0.03);<br>negative regulation of interferon-beta production (GO:0032688; FDR=0.035);<br>cellular response to transforming growth factor beta stimulus (GO:0071560; FDR=0.02);<br>cellular response to cytokine stimulus (GO:0071345; FDR=0.008);<br>regulation of apoptotic process (GO:0042981; FDR=0.01)

1 – reference (Ref) / alternative (SNP) allele;
2 – TF - transcription factor;
3 – binding of TF to the reference (LOSS) / alternative (GAIN) allele;
4 – binding sites with high affinity for TF;
5 – p value statistically confirming the potential gain or loss of function of the genomic region with SNP in terms of transcription factor binding;
6 – p-value for assessing the binding of TF to the Ref allele;
7 – p-value for assessing the binding of TF to the SNP allele;
8 – biological processes pathogenetically significant for IS, in which TFs that bind to the alternative allele are jointly involved (data from the Gene Ontology resource; http://geneontology,org/);
9 – biological processes pathogenetically significant for AI, in which TFs that bind to an reference allele are jointly involved (data from the Gene Ontology resource; http://geneontology,org/)

Table S7: Analysis of the effect of rs1136141 *HSPA8* on binding of DNA to transcription factors

№	Ref/ SNP allele[1]	TF[2]	GAIN /LOSS[3]	Motif[4]	P-Value SNP impact[5]	P-Value Ref[6]	P-Value SNP[7]
1	G/A	PAX4	gain	PAX4_1	1.2×10^{-6}	5.9×10^{-6}	0.165
2	G/A	HEY1	gain	HEY1_disc2	4.5×10^{-5}	0.001	0.307
3	G/A	PAX4	gain	PAX4_3	0.001	0.008	0.958
4	G/A	RREB1	gain	RREB1_1	0.001	0.019	0.184
5	G/A	GLIS1	gain	GLIS1_1	0.002	0.024	0.535
6	G/A	KLF4	gain	KLF4_1	0.002	0.005	0.085
7	G/A	RUNX2	gain	RUNX2_6	0.003	0.021	0.467
8	G/A	RREB1	gain	RREB1_2	0.003	0.041	0.184
9	G/A	KLF16	gain	KLF16_1	0.003	0.026	0.454
10	G/A	EBF1	gain	EBF1_1	0.004	0.011	0.077
11	G/A	TATA	gain	TATA_disc4	0.004	0.005	0.088
12	G/A	Klf4	gain	MA0039.2	0.004	0.007	0.089
13	G/A	NR2C2	gain	NR2C2_disc3	0.005	0.012	0.138
14	G/A	TBX5	gain	TBX5_1	0.005	0.015	0.319
15	G/A	IRF	gain	IRF_disc4	0.005	0.029	0.415
16	G/A	NR2F2	gain	NR2F2_1	0.005	0.034	0.207
17	G/A	SP1	gain	SP1_7	0.007	0.015	0.224
18	G/A	EBF1	gain	EBF1_4	0.008	0.009	0.100
19	G/A	KLF5	gain	MA0599.1	0.009	0.003	0.058
20	G/A	PPARA	gain	PPARA_1	0.011	0.012	0.059
21	G/A	TBX1	gain	TBX1_1	0.012	0.027	0.213
22	G/A	ZNF281	gain	ZNF281_1	0.012	0.038	0.228
23	G/A	RUNX3	gain	RUNX3_4	0.012	0.033	0.436
24	G/A	RUNX1	gain	RUNX1_7	0.013	0.035	0.376
25	G/A	EGR1	gain	EGR1_disc6	0.015	0.023	0.217
26	G/A	EGR1	gain	EGR1_disc5	0.017	0.033	0.156
27	G/A	RUNX3	gain	RUNX3_2	0.018	0.029	0.389

28	G/A	WT1	gain	WT1_1	0.018	0.041	0.263
29	G/A	SP1	gain	SP1_2	0.018	0.029	0.188
30	G/A	RUNX1	gain	RUNX1_9	0.019	0.044	0.445
31	G/A	CTCF	gain	CTCF_disc6	0.019	0.007	0.087
32	G/A	ATF3	gain	ATF3_disc3	0.021	0.031	0.276
33	G/A	SP1	gain	SP1_4	0.026	0.031	0.173
34	G/A	KLF12	gain	KLF12_2	0.028	0.024	0.149
35	G/A	EGR3	gain	EGR3_3	0.031	0.024	0.119
36	G/A	KLF7	gain	PAX4_1	1.2×10^{-6}	5.9×10^{-6}	0.165
37	G/A	RREB1	gain	HEY1_disc2	4.5×10^{-5}	0.001	0.307
38	G/A	ELF5	gain	PAX4_3	0.001	0.008	0.958
39	G/A	GLIS2	gain	RREB1_1	0.001	0.019	0.184
40	G/A	ETS1	gain	GLIS1_1	0.002	0.024	0.535
41	G/A	TFAP2	gain	KLF4_1	0.002	0.005	0.085
42	G/A	E4F1	gain	RUNX2_6	0.003	0.021	0.467
43	G/A	SP1	gain	RREB1_2	0.003	0.041	0.184
44	G/A	TEAD1	loss	MA0090.1	1.5×10^{-4}	0.108	0.007
45	G/A	FEV	loss	FEV_1	0.001	0.169	0.006
46	G/A	EGR1	loss	EGR1_disc4	0.001	0.094	0.014
47	G/A	TEAD1	loss	TEAD1_2	0.002	0.086	0.005
48	G/A	IKZF2	loss	IKZF2_1	0.005	0.519	0.024
49	G/A	TEAD1	loss	TEAD1_1	0.006	0.144	0.006
50	G/A	CREB1	loss	CREB1_8	0.007	0.517	0.021
51	G/A	E2F1	loss	E2F1_10	0.009	0.306	0.020
52	G/A	TEAD3	loss	TEAD3_2	0.009	0.284	0.019
53	G/A	STAT	loss	STAT_disc1	0.009	0.313	0.039
54	G/A	Stat5a::Stat5b	loss	MA0519.1	0.010	0.195	0.017
55	G/A	TEAD1	loss	TEAD1_3	0.011	0.264	0.014
56	G/A	GATA2	loss	GATA2_2	0.019	0.238	0.019
57	G/A	IKZF1	loss	IKZF1_1	0.020	0.360	0.047
58	G/A	NFATC1	loss	NFATC1_3	0.026	0.128	0.023
59	G/A	ETS	loss	MA0090.1	1.5×10^{-4}	0.108	0.007
60	G/A	ZNF524	loss	FEV_1	0.001	0.169	0.006
61	G/A	ZNF589	loss	EGR1_disc4	0.001	0.094	0.014
62	G/A	BCL	loss	TEAD1_2	0.002	0.086	0.005
	A[8]	positive regulation of CD8-positive, alpha-beta T cell differentiation (GO:0043378; FDR=0.007);<br>regulation of blood vessel endothelial cell migration (GO:0043535; FDR=0.04);<br>positive regulation of angiogenesis (GO:0045766; FDR=0.02);<br>response to growth factor (GO:0070848; FDR=0.015);<br>negative regulation of apoptotic process (GO:0043066; FDR=0.04)					
	G[9]	-					

1 – reference (Ref) / alternative (SNP) allele;
2 – TF - transcription factor;
3 – binding of TF to the reference (LOSS) / alternative (GAIN) allele;
4 – binding sites with high affinity for TF;
5 – p value statistically confirming the potential gain or loss of function of the genomic region with SNP in terms of transcription factor binding;
6 – p-value for assessing the binding of TF to the Ref allele;
7 – p-value for assessing the binding of TF to the SNP allele;
8 – biological processes pathogenetically significant for IS, in which TFs that bind to the alternative allele are jointly involved (data from the Gene Ontology resource; http://geneontology,org/);

9 – biological processes pathogenetically significant for AI, in which TFs that bind to an reference allele are jointly involved (data from the Gene Ontology resource; http://geneontology,org/)
